# SAMURAI: Sensitivity analysis of a meta-analysis with unpublished but registered analytical investigations (software)

**DOI:** 10.1186/2046-4053-3-27

**Published:** 2014-03-18

**Authors:** Noory Y Kim, Shrikant I Bangdiwala, Kylie Thaler, Gerald Gartlehner

**Affiliations:** 1 Department of Biostatistics, University of North Carolina, Chapel Hill, NC, 27599, USA; 2 Department for Evidence-based Medicine and Clinical Epidemiology, Danube University, Krems, Austria; 3 Research Triangle Institute-International, Research Triangle Park, NC, USA

**Keywords:** Clinical trial registries, Meta-analysis, Publication bias, R-software, Sensitivity analysis, Statistical software, Unpublished studies

## Abstract

**Background:**

The non-availability of clinical trial results contributes to publication bias, diminishing the validity of systematic reviews and meta-analyses. Although clinical trial registries have been established to reduce non-publication, the results from over half of all trials registered in ClinicalTrials.gov remain unpublished even 30 months after completion. Our goals were i) to utilize information available in registries (specifically, the number and sample sizes of registered unpublished studies) to gauge the sensitivity of a meta-analysis estimate of the effect size and its confidence interval to the non-publication of studies and ii) to develop user-friendly open-source software to perform this quantitative sensitivity analysis.

**Methods:**

The open-source software, the R package SAMURAI, was developed using R functions available in the R package metafor. The utility of SAMURAI is illustrated with two worked examples.

**Results:**

Our open-source software SAMURAI, can handle meta-analytic datasets of clinical trials with two independent treatment arms. Both binary and continuous outcomes are supported. For each unpublished study, the dataset requires only the sample sizes of each treatment arm and the user predicted ‘outlook’ for the studies. The user can specify five outlooks ranging from ‘very positive’ (i.e., very favorable towards intervention) to ‘very negative’ (i.e., very favorable towards control).

SAMURAI assumes that control arms of unpublished studies have effects similar to the effect across control arms of published studies. For each experimental arm of an unpublished study, utilizing the user-provided outlook, SAMURAI randomly generates an effect estimate using a probability distribution, which may be based on a summary effect across published trials. SAMURAI then calculates the estimated summary treatment effect with a random effects model (DerSimonian & Laird method), and outputs the result as a forest plot.

**Conclusions:**

To our knowledge, SAMURAI is currently the only tool that allows systematic reviewers to incorporate information about sample sizes of treatment groups in registered but unpublished clinical trials in their assessment of the potential impact of publication bias on meta-analyses. SAMURAI produces forest plots for visualizing how inclusion of registered unpublished studies might change the results of a meta-analysis. We hope systematic reviewers will find SAMURAI to be a useful addition to their toolkit.

## Background

**Figure 1:**
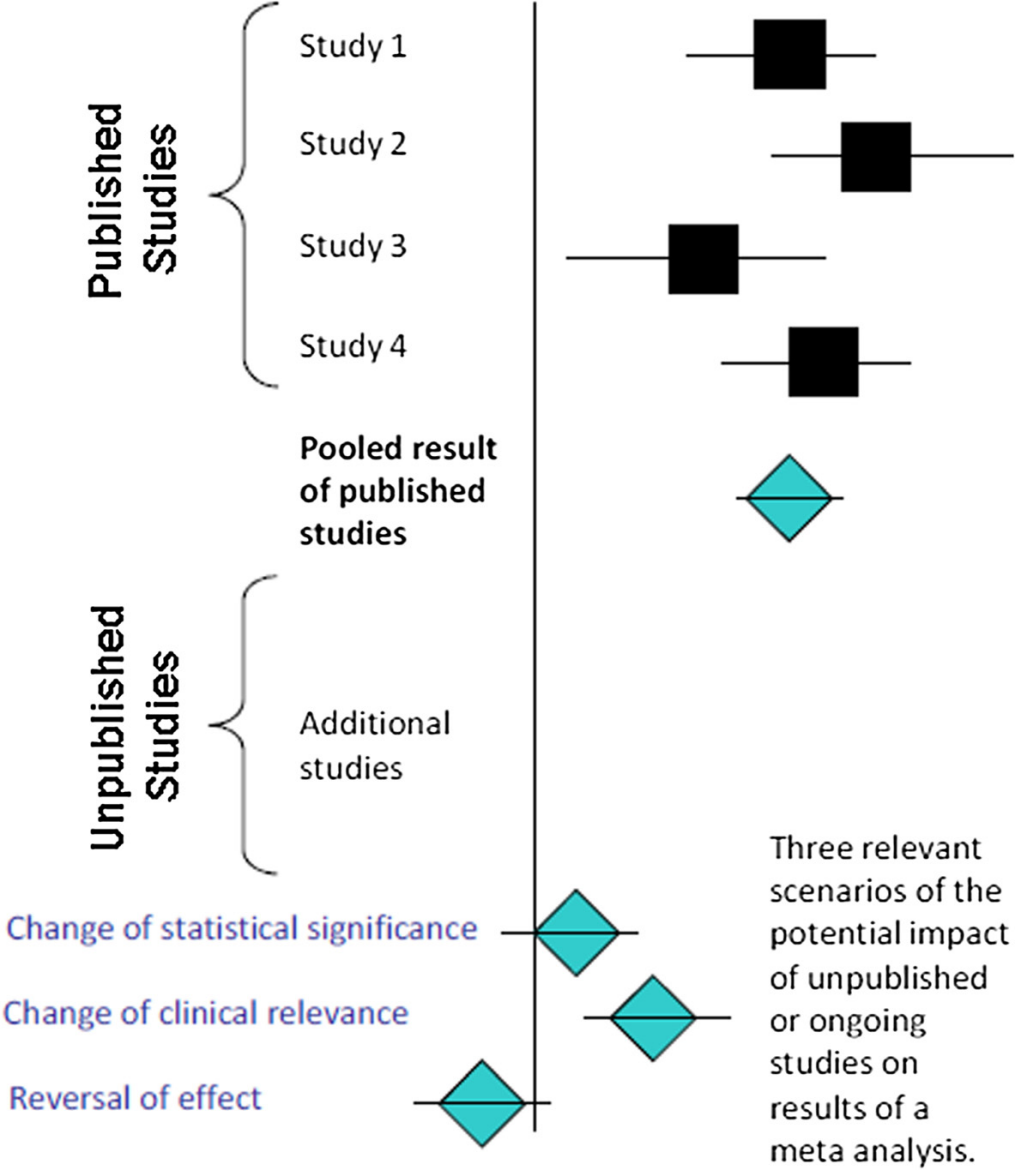
**Schematic illustration of the potential impact of unpublished studies on a meta-analytic summary effect.** The vertical line demarcates an effect size showing no difference between treatment groups for the outcome being studied. In this case, effect sizes to the right of the vertical line correspond to the intervention treatment group having more favorable outcomes than the control treatment group; all four published studies found the intervention treatment to be significantly better than the control treatment with respect to the study outcome, as indicated by having confidence intervals completely to the right of the vertical line. The first diamond shows the pooled estimate across the four published studies. If we then include results from unpublished studies in the meta-analysis, how might the conclusion of the meta-analysis change? The three diamonds at the bottom of the figure each represent a possible outcome of the pooled estimate across all studies (published and unpublished).

Clinicians, policy makers, and patients rely on the results of clinical trials to make informed decisions about health care. Meta-analyses collate and combine results of clinical trials to provide a quantitative summary of available evidence regarding a specific clinical question. Unfortunately, the non-publication of trial results can undermine the ability of meta-analysts to accurately estimate a summary treatment effect [1]. In particular, the non-release of negative or non-significant results may lead to the overestimation of the magnitude of a treatment effect and consequently to false conclusions about the treatment’s efficacy (Figure 1).

For example, a systematic review by Jefferson et al. [2] estimated that 60 percent of patient data from 10 Roche-conducted trials of oseltamivir (sold as ‘Tamiflu’) remained unavailable to reviewers even after two years, despite repeated requests for the data [3]. This high percentage of unavailable information is clearly inadequate for making robust conclusions about the efficacy and the risks of using the drug.

Recent developments to mitigate publication bias due to the non-publication of studies include i) the formation of public clinical trial registries for the prospective registration of clinical trials, such as ClinicalTrials.gov in the USA and ClinicalTrialsRegister.eu in the European Union, ii) the adoption of a policy by the International Committee of Medical Journal Editors (ICMJE) in 2005 that the journals they oversee would only publish results of clinical trials which have been prospectively registered in a public registry [4], and iii) the passage of the Food and Drug Administration Amendments Act (FDAAA) of 2007 which expanded the requirements for the registry of clinical trials that receive USA federal funding. These measures have led to a dramatic increase in the number and proportion of clinical trials that are prospectively registered [5]. However, researchers are not necessarily obligated to release to the public the results of such trials. Ross et al. [6] found that in a sample of NIH-funded trials registered with ClinicalTrials.gov, less than half were published within 30 months of trial completion. Thus, while systematic reviewers may be aware of trials that have been conducted and have details regarding trial methodologies and sample sizes, they may not have timely access to the results to include them in a meta-analysis.

Our goal was to design and program a pragmatic exploratory tool for the systematic reviewer who wishes to visualize the sensitivity of a meta-analytic summary to the addition of one or more registered, unpublished studies (RUSTs). We wanted our software to be open source, easy to use, and with output easy to understand. Our result, which we introduce in this paper, is the R package Sensitivity Analysis of a Meta-analysis with Unpublished but Registered Analytical Investigations (SAMURAI).

Statistical methods used for gauging the potential impact of publication bias, such as the trim and fill method [7] and the Copas selection model [8], already exist. As far as we know, however, no existing method uses the information available in clinical trial registries to conduct the sensitivity of a meta-analytic summary effect to RUSTs. Therefore, we believe that SAMURAI can be a useful addition to the systematic reviewer’s toolkit.

## Methods

### Assumptions

In developing the software application, we assumed the following:

i) The meta-analysis consists of randomized clinical trials, all addressing the same research question and each with two independent intervention arms; an experimental arm and a control arm. ii) For each RUST, the clinical trial registry has information on the sample sizes of both treatment arms. iii) Effect rates in the control arms of RUSTs are the same as the pooled event rate across control arms of all published studies in the meta-analysis. iv) Any variation among studies is adequately accounted for by random effects models; no additional information on covariates is necessary in order to explain this variation.

We make no assumptions about the effect size of the experimental arms of RUSTs. Instead, we leave it up to the end-user to decide the anticipated effect size and direction of each RUST. In essence, with our methodology, the end-user acquires the added flexibility and responsibility of predicting anticipated effect sizes and directions.

### Description of algorithm for the computer software

The software requires that all published studies in the meta-analytic dataset report their results in a similar format. Either all the studies have binary outcomes or all the studies have continuous outcomes. Studies with binary outcomes should report 2 × 2 tables. Studies with continuous outcomes should all report their results as either i) the mean effect size and its standard deviation within each treatment arm, or ii) the standardized mean difference.

The dataset should also have the sample sizes of both treatment arms for every study, including RUSTs. Entries in ClinicalTrials.gov typically indicate total sample size but not sample sizes for each treatment arm. We recommend simply assuming a 1:1 allocation ratio between the two treatment arms, unless the entry specifies a different allocation ratio.

The end-user assigns each RUST an ‘outlook’ specifying the size and direction of the effect. The end-user can choose outlooks from a list of predefined options ranging from ‘very positive’, for which the RUST is anticipated to heavily favor the experimental intervention, to ‘very negative’, for which the RUST is anticipated to heavily favor the control intervention.

We defined ten such outlooks for RUSTs. For five of these outlooks, the summary effect is associated with a fixed number (which is preset to a default value but can be adjusted by the end-user), whereas the other five outlooks are based on the summary effect across the published studies or its confidence interval.

With binary-outcome studies, SAMURAI will impute the relative risk of each RUST according to the outlook assigned to it by the end-user (Table 1). With studies having continuous outcomes in the form of means and standard deviations for each arm, the data are converted to standardized mean differences (SMD). For each RUST, SAMURAI will impute a SMD according to the outlook assigned to it by the end-user (Table 2).

**Table 1:** Default risk ratios (RR) assigned to unpublished studies with binary outcomes

**Outlook**	**Depends on published studies?**	**If the outcome event is desired (higher.is.better = T)**	**If the outcome event is undesired (higher.is.better = F)**
‘very positive’	No	3	0.33
‘positive’		2	0.5
‘no effect’		1	1
‘negative’		0.5	2
‘very negative’		0.33	3
‘very positive CL’	Yes, depends on CL of published studies	*UCL*_*pub*,1-*α*_	*LCL*_*pub*,1-*α*_
‘positive CL’		$0.5\;\left({\hat{\mathrm{RR}}}_{\mathrm{pub}}+\mathrm{UC}L_{\mathrm{pub},1a}\right)$	$0.5\;\left({\hat{\mathrm{RR}}}_{\mathrm{pub}}+\mathrm{LC}L_{\mathrm{pub},1a}\right)$
‘current effect’		${\hat{\mathrm{RR}}}_{\mathrm{pub}}$	${\hat{\mathrm{RR}}}_{\mathrm{pub}}$
‘negative CL’		$0.5\;\left({\hat{\mathrm{RR}}}_{\mathrm{pub}}+\mathrm{LC}L_{\mathrm{pub},1-a}\right)$	$0.5\;\left({\hat{\mathrm{RR}}}_{\mathrm{pub}}+\mathrm{UC}L_{\mathrm{pub},1-a}\right)$
‘very negative CL’		*LCL*_*pub*,1-*α*_	*UCL*_*pub*,1-*α*_

Note: ‘Positive’ outlooks favor the experimental treatment arm and ‘negative’ outlooks favor the control treatment arm.

RR: Risk ratio, or relative risk.

LCL: Lower confidence limit.

UCL: Upper confidence limit.

${\hat{\mathrm{RR}}}_{\mathrm{pub}}$ : The summary RR across all published studies.

*LCL*_*pub*,1-*α*_,*UCL*_*pub*,1-*α*_: The 1-*α* confidence interval of ${\hat{\mathrm{RR}}}_{\mathrm{pub}}$.

**Table 2:** Default standardized mean differences (SMD) assigned to unpublished studies with continuous outcomes

**Outlook**	**Depends on published studies?**	**If the outcome event is desired (higher.is.better = T)**	**If the outcome event is undesired (higher.is.better = F)**
‘very positive’	No	0.8	−0.8
‘positive’		0.3	−0.3
‘no effect’		0	0
‘negative’		−0.3	0.3
‘very negative’		−0.8	0.8
‘very positive CL’	Yes, depends on CL of published studies	*UCL,*_*pub*,1-*α*_	*LCL,*_*pub*,1-*α*_
‘positive CL’		$0.5\left({\hat{\mathrm{SMD}}}_{\mathrm{pub}}+\mathrm{UC}L_{\mathrm{pub},1-\alpha }\right)$	$0.5\left({\hat{\mathrm{SMD}}}_{\mathrm{pub}}+\mathrm{LC}L_{\mathrm{pub},1-\alpha }\right)$
‘current effect’		${\hat{\mathrm{SMD}}}_{\mathrm{pub}}$	${\hat{\mathrm{SMD}}}_{\mathrm{pub}}$
‘negative CL’		$0.5\left({\hat{\mathrm{SMD}}}_{\mathrm{pub}}+\mathrm{LC}L_{\mathrm{pub},1-\alpha }\right)$	$0.5\left({\hat{\mathrm{SMD}}}_{\mathrm{pub}}+\mathrm{UC}L_{\mathrm{pub},1-\alpha }\right)$
‘very negative CL’		*LCL,*_*pub*,1-*α*_	*UCL,*_*pub*,1-*α*_

Note: ‘Positive’ outlooks favor the experimental treatment arm and ‘negative’ outlooks favor the control treatment arm.

SMD: Standardized mean difference.

LCL: Lower confidence limit.

UCL: Upper confidence limit.

${\hat{\mathrm{SMD}}}_{\mathrm{pub}}$ : The summary SMD across all published studies.

(*LCL*_*pub*,1-*α*_, *UCL*_*pub*,1-*a*_): The 1-α confidence interval of ${\hat{\mathrm{SMD}}}_{\mathrm{pub}}$.

In addition, SAMURAI will impute the variance of the SMD of a RUST using Borenstein’s *ad-hoc* ‘very good’ approximation [9]. We chose Borenstein’s approximation as a matter of convenience, since it requires only the sample sizes of the treatment arms in the unpublished studies and the summary SMD across the published studies, thereby bypassing the need to impute variances for each treatment arm of a RUST.

Based on the end-user’s outlook selection for a RUST, the software imputes an effect size and confidence interval for that RUST, using the predefined effect size associated with that outlook, along with random noise thrown in to mirror uncertainty in the estimate of the non-published effect size. Once the effect sizes and confidence intervals of the RUST are imputed, the software calculates a summary effect using standard meta-analysis methods. By default, the software generates a random effects model using the popular inverse-variance method by DerSimonian and Laird [10]. More mathematical details can be found in Additional file 1, which contains pseudo-algorithmic descriptions of the methodology used by SAMURAI.

We opted to use a random effects model since it allows statistical inferences to be made about studies other than those in the meta-analysis. In contrast, statistical inferences made using a fixed-effects model are limited to the studies in the meta-analysis [11].

The software outputs the results as forest plots. Each forest plot includes a summary effect across just the published studies, a summary effect across just the unpublished studies (whose outlooks are chosen by the end-user), and a summary effect across all the studies, published and unpublished, along with the between-study variance τ^2^.

### Software

We developed our software as an R package instead of as a spreadsheet program for the following reasons: i) R is a widely used and freely available language for statistical computing, and ii) R can produce better-looking and more consistent graphics than a spreadsheet program. In addition, R can readily be used to export graphics to an Adobe Portable Document Format (PDF) file.

Learning to use R may be more difficult than learning how to use a spreadsheet program; however, there are a number of tutorials available online or in print form. We hope that end-users unfamiliar with R will find the worked examples in this article a helpful primer.

### Results and worked examples

SAMURAI is available free of charge and it is open-source. The program and code are freely available on the Internet via the Comprehensive R Archive Network (CRAN) at http://cran.r-project.org/web/packages/SAMURAI/index.html. SAMURAI employs functions in the R package metafor[12].

### Installing and running SAMURAI

The end-user will first need to install R on their computer, available through CRAN at http://cran.r-project.org. R is available for computers with Linux, Macintosh, or Windows operating systems. Once the end-user has installed R and can run R on their computer, they can install SAMURAI with the following command:

> install.packages(‘SAMURAI’)

(Note that the caret > represents a prompt that is not typed by the end-user.)

Then the end-user can begin using **SAMURAI** after typing in the following command:

> library(SAMURAI)

### Formatting of dataset files

End-users of SAMURAI will first need to prepare their dataset as a comma separated value (CSV) file with specific column headings. This data file can be created in a spreadsheet program such as the open source LibreOffice Calc or Microsoft Office Excel, then imported into R. Details can be found in Additional file 1.

### Worked example 1: trials with binary outcomes

We consider a dataset of trials published from 1990 to 2001 comparing counts of non-healing of duodenal ulcer in patients on ulcer-healing drug with *H. pylori* eradication treatment (experimental arm) versus counts of non-healing in patients on ulcer-healing drug alone (control arm). The example dataset consists of 33 published studies listed in a meta-analysis by Ford et al. [13] (Table 3).

**Table 3:** **The dataset**Hpylori**, which is included in the R package SAMURAI**

**Study number**	**Study name**	**Year**	**Outlook**	**expt.events**	**expt.n**	**ctrl.events**	**ctrl.n**
1	Rauws	1990	published	7	24	5	26
2	Graham	1991	published	4	53	10	52
3	Bayrerdorffer	1992	published	2	29	4	29
4	Hosking	1992	published	8	78	21	77
5	Bianchi Porro	1993	published	7	91	12	92
6	Hentschel	1993	published	1	52	3	52
7	Mantzaris	1993	published	5	17	8	16
8	Wang	1993	published	3	23	6	36
9	Lin	1994	published	0	21	2	21
10	Spinzi	1994	published	2	24	3	29
11	Bayrerdorffer	1995	published	4	136	12	128
12	Katoh	1995	published	0	27	1	25
13	Logan	1995	published	2	70	6	78
14	Pinero	1995	published	8	30	7	30
15	Sobhani	1995	published	7	59	15	60
16	Avsar	1996	published	2	23	10	22
17	Figueroa	1996	published	4	57	4	43
18	Harford	1996	published	36	127	25	69
19	Kato	1996	published	0	28	1	23
20	O'Morain	1996	published	9	102	15	106
21	Parente	1996	published	7	63	1	33
22	Porro	1996	published	2	17	9	15
23	Shirotani	1996	published	4	25	6	25
24	Bardhan	1997	published	4	141	6	74
25	Carpintero	1997	published	3	78	3	44
26	Pounder	1997	published	5	61	8	30
27	Graham	1998	negative	NA	77	NA	76
28	Schwartz	1998	positive	NA	292	NA	60
29	Kepecki	1999	negative	NA	39	NA	34
30	van Zanten	1999	very positive	NA	98	NA	48
31	Wong	1999	negative	NA	57	NA	57
32	Asaka	2001	positive	NA	205	NA	51
33	Mones	2001	positive	NA	42	NA	43

expt.n: Sample size of the experimental treatment arm.

ctrl.n: Sample size of the control/reference treatment arm.

expt.events: Number of events occurring within the experimental treatment group.

ctrl.events: Number of events occurring within the control/reference treatment group.

For the purposes of illustration, we will pretend that a subset of these studies were registered but never published, i.e., RUSTs. For each RUST, we remove information on the number of events, but leave in the sample sizes.

A version of the dataset is included in the SAMURAI package as the dataset Hpylori, which differs from the original dataset in Ford et al. [13] in that seven of the 33 studies are treated as unpublished. The end-user may load and view this modified dataset by typing in the following commands at the R prompt:

> data(Hpylori)

> Hpylori

The second of these commands displays the dataset.

The sample sizes of the control and experimental arms are in the columns labeled ctrl.n and expt.n, respectively. The number of events in the control and experimental arms are in the columns labeled ctrl.events and expt.events, respectively.

To generate a forest plot (not shown) for the dataset, we can give the following command:

> forestsens(table=Hpylori, binary=TRUE, higher.is.better=FALSE, scale=0.8)

For reproducible results, one may freeze the random variation by specifying a seed for the random number generator, as in the following example:

> forestsens(table=Hpylori, binary=TRUE, higher.is.better=FALSE, scale=0.8, random.number.seed=106)

With random.number.seed=106 the summary relative risk across all 33 studies is 0.64, with a 95% confidence interval of (0.49, 0.82) (Figure not shown). End-users may get slightly different results depending on which, if any, random number seed they specify.

Since the outcome being measured is binary, we specified binary=TRUE. Furthermore, the events in the dataset indicate the number of patients not healed; we thereby specify higher.is.better=FALSE. Specifying the scale parameter is optional, but in this case helps our plot look neater by reducing font sizes to 80% of the default font size.

Suppose we want to modify the outlooks of all unpublished studies to, say, ‘very negative’. We can do this with the following command:

> forestsens(table=Hpylori, binary=TRUE, higher.is.better=FALSE, scale=0.8, random.number.seed=106,**outlook=‘very negative’**)

**Figure 2:**
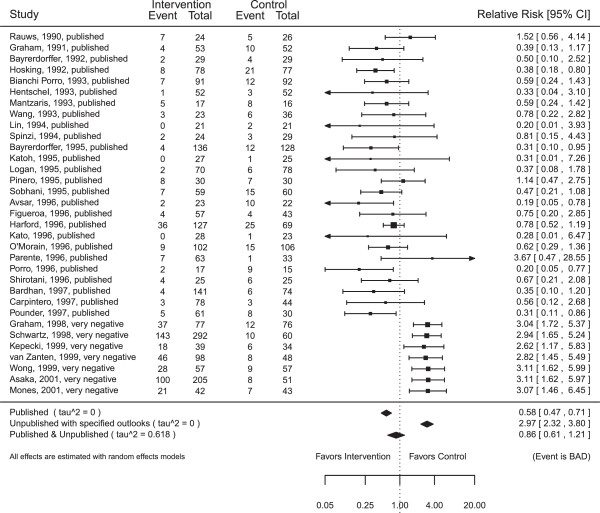
**Forest plot for the dataset**Hpyloris**with all seven unpublished studies assigned ‘very negative’ outcomes.** The dataset has 33 published studies. The random number seed used to generate this figure was 106.

In order to illustrate how an end-user would actually examine the effect of RUSTs and their assumptions of outlooks on the summary effect size, Figure 2 presents the forest plot output from SAMURAI when the last seven studies are considered as RUSTs and the outlook chosen for the ‘unpublished’ studies is ‘very negative’. We see that the effect size now does not significantly differ from 1.

Note that in Figure 2, the relative risks for unpublished studies have values that are close to but not exactly 3, which is the relative risk value assigned to ‘very negative’ studies with binary events for ‘bad’ outcome variables.

**Figure 3:**
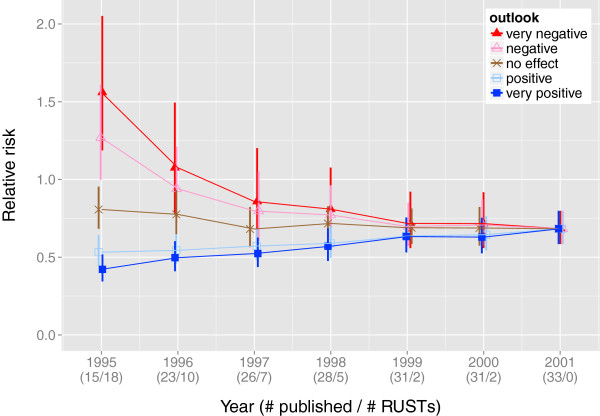
**Sensitivity analysis using the dataset**Hpylori. This figure shows the estimated summary effect sizes for the dataset Hpylori with corresponding 95% confidence intervals, as the number of unpublished studies is diminished and with all RUSTs having the same outlook, ranging from ‘very positive’ to ‘very negative’. This figure was prepared with the R package *ggplot2*.

As a sensitivity analysis, we compare the summary effect sizes and their 95% confidence intervals under different scenarios. For the purposes of illustration, we pretend that varying subsets of these studies were registered but never published; using the original dataset in Ford et al. [13], we set studies published from 1995 onwards as RUSTs and incrementally consider as published the studies in subsequent years, until reaching 2001. We then create a sensitivity plot of effect sizes as more studies are considered published. We examine the scenarios under which all RUSTs are assigned the same outlook, ranging from ‘very positive’ to ‘very negative’. Figure 3 displays the variation in the estimated effect sizes as studies from 1995 onwards are considered published or not.

One can generate a forest plot for each of the ten outlooks by specifying the option all.outlooks=TRUE, which will assign the same outlook to all RUSTs:

> forestsens(table=Hpylori, binary=TRUE, higher.is.better=FALSE, scale=0.8, random.number.seed=106,**all.outlooks=TRUE**)

We can put all of these plots into a single PDF file (in this case with the name filename.pdf) with the following commands:

> pdf(‘filename.pdf’)

> forestsens(table=Hpylori, binary=TRUE, higher.is.better=FALSE, scale=0.8, random.number.seed=106,**all.outlooks=TRUE**)

> dev.off()

When the parameter all.outlooks=TRUE is specified, the function forestsens also generates a table of overall summary effects, their confidence intervals (from the lower confidence limit lcl to the upper confidence limit ucl), and an estimate of τ^2^ (tau2), which is a measure of heterogeneity between studies.

The forestsens function allows the end-user to override the default relative risk assigned to any outlook. For example, if we want to change the relative risks of ‘very negative’ studies from the default of 3 to 2.5, and to change the relative risks of ‘negative’ studies from 2 to 1.5, we can do so as follows:

> forestsens(table=Hpylori, binary=TRUE, higher.is.better=FALSE, scale=0.8, random.number.seed=106, all.outlooks=TRUE,**rr.neg=1.5, rr.vneg=2.5**)

The results are included in Table 4.

**Table 4:** **Summary effects for the**Hpylori**dataset after all unpublished studies assigned the same default outlook**

**Scenario number**	**Outlook assigned to all 7 unpublished studies**	**Relative risk (RR) assigned to all unpublished studies**	**Summary effect (RR)**	**Lower 95% confidence limit**	**Upper 95% confidence limit**	**τ**^2^**
1	very positive	0.33	0.518	0.432	0.621	<0.001
2	positive	0.5	0.561	0.471	0.669	<0.001
3	no effect	1	0.697	0.585	0.829	0.011
4	negative	2	0.801	0.609	1.052	0.317
4a*	negative	1.5	0.759	0.602	0.958	0.166
5	very negative	3	0.866	0.616	1.217	0.636
5a*	very negative	2.5	0.841	0.614	1.151	0.501
6	very positive CL	*LCL*_*pub*,1-*α*_	0.546	0.457	0.652	<0.001
7	positive CL	$0.5\left({\hat{\mathrm{RR}}}_{\mathrm{pub}}+\mathrm{LC}L_{\mathrm{pub},1-\alpha }\right)$	0.573	0.481	0.682	<0.001
8	current effect	${\hat{\mathrm{RR}}}_{\mathrm{pub}}$	0.591	0.497	0.703	<0.001
9	negative CL	$0.5\left({\hat{\mathrm{RR}}}_{\mathrm{pub}}+\mathrm{UC}L_{\mathrm{pub},1-\alpha }\right)$	0.603	0.507	0.716	<0.001
10	very negative CL	*UCL*_*pub*,1-*α*_	0.634	0.534	0.752	<0.001

(The random number seed used to generate this table was 106.)

*In these cases all unpublished studies were assigned an RR different from the default RR.

**A displayed value of ‘0’ for ‘tau2’ indicates a τ^2^ value less than 0.001.

### Worked example 2: trials with continuous outcomes

A systematic review by Jurgens et al. [14] included a meta-analysis of the effect of green tea consumption on weight loss using 14 placebo-controlled randomized trials published between 2004 and 2010.

Of these fourteen studies, we shall, for the purposes of our example, arbitrarily treat the three studies published from 2009 onward as RUSTs. Thus the dataset greentea, included in the SAMURAI package, contains 11 published studies and three RUSTs (Table 5). This dataset can be loaded into memory and viewed by typing in the following commands:

**Table 5:** **The dataset**greentea**, which is included in the R package SAMURAI**

**Study number**	**Study name**	**Year**	**Outlook**	**expt.mean**	**expt.sd**	**expt.n**	**ctrl.mean**	**ctrl.sd**	**ctrl.n**
1	Kataoka	2004	published	−1.3	1.7	71	−0.8	1.7	71
2	Takashima	2004	published	−1.6	1.9	10	−1.4	1.5	9
3	Diepvens	2005	published	−4.21	2.7	23	−4.19	1.3	23
4	Kajimoto	2005	published	−0.55	2.1	129	0.6	2	66
5	Kozuma	2005	published	−2.7	1.5	107	0.8	0.9	119
6	Hill	2007	published	0.08	0.9	19	−0.45	1.2	19
7	Nagao	2007	published	−1.7	1.5	123	−0.1	1.7	117
8	Auvichayapat	2008	published	−2.7	2.2	30	−2	9.7	30
9	Hsu	2008	published	−0.15	2	41	−0.03	1.9	37
10	Takase	2008	published	−2.9	1.2	44	0.1	0.7	45
11	Takeshita	2008	published	−1.1	1.3	40	−0.3	1.5	41
12	Maki	2009	positive	NA	NA	65	NA	NA	63
13	Suzuki	2009	no effect	NA	NA	18	NA	NA	20
14	Wang	2010	no effect	NA	NA	139	NA	NA	43

expt.n: Sample size of the experimental treatment arm.

ctrl.n: Sample size of the control/reference treatment arm.

expt.mean: Mean effect size within the experimental treatment group.

ctrl.mean: Mean effect size within the control/reference treatment group.

expt.sd: Standard deviation of effect size within the experimental treatment group.

ctrl.mean: Standard deviation of effect size within the control/reference treatment group.

> data(greentea)

> greentea

The sample sizes of the control and experimental arms are in the columns labeled ctrl.n and expt.n, respectively. The mean weight loss in control and experimental arms are in the columns labeled ctrl.mean and expt.mean, respectively. Their respective standard deviations are ctrl.sd and expt.sd.

We can generate a forest plot for the dataset with the following command:

> forestsens(greentea, binary=FALSE, mean.sd=TRUE, higher.is.better=FALSE)

Since the outcome being measured is continuous (and hence not binary), we specified binary=FALSE. Furthermore, since the outcome data are in the form of means and standard deviations, we specify mean.sd=TRUE.

In this example, a more negative change in weight is desired. That is to say, we desire that the weight (outcome) in the experimental arm will be lower than the weight (outcome) in the control arm. When a lower outcome is desired, as in this case, choose the option higher.is.better=FALSE (conversely, if a higher outcome is desired, choose the option higher.is.better=TRUE).

Again, to make these results reproducible, we can specify a random.number.seed with any integer, as in the following example:

> forestsens(greentea, binary=FALSE, mean.sd=TRUE, higher.is.better=FALSE,**random.number.seed=52**)

Suppose we want to modify the outlooks of all unpublished studies to, say, ‘negative’. We can do this with the following command:

> forestsens(greentea, binary=FALSE, mean.sd=TRUE, higher.is.better=FALSE,random.number.seed=52,**outlook=‘negative’**)

**Figure 4:**
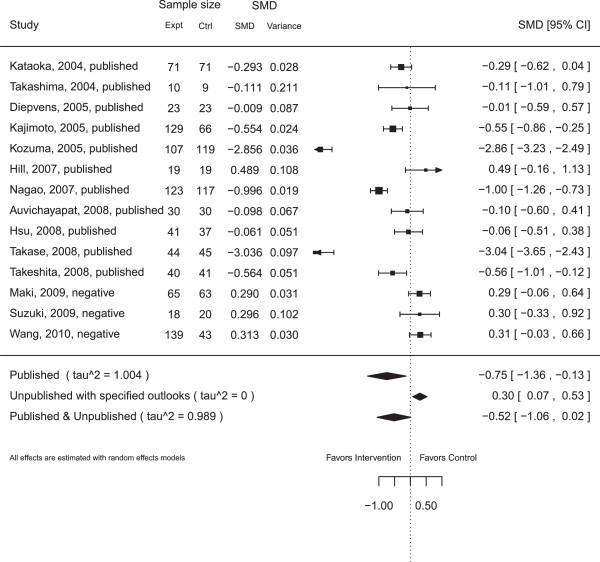
**Forest plot for the dataset**greentea** with all three unpublished studies assigned to have ‘negative’ outcomes.** The dataset has 11 published studies. The random number seed used to generate this figure was 52.

Note that in Figure 4, the SMD for each unpublished study is close to but not exactly 0.3, which is the SMD assigned to ‘negative’ studies when a lower outcome is preferable.

**Figure 5:**
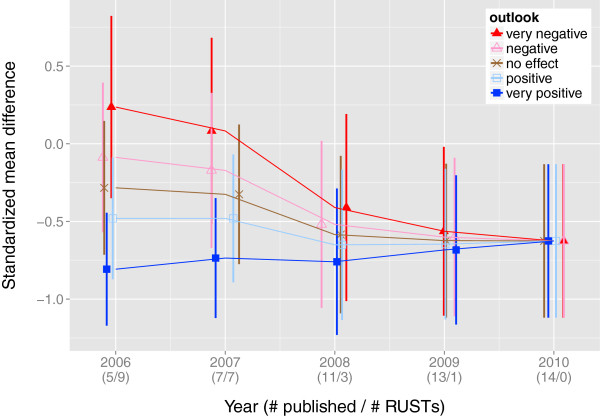
**Sensitivity analysis using the dataset**greentea. The figure shows the estimated summary effect sizes for the dataset greentea with corresponding 95% confidence intervals, as the number of unpublished studies is diminished and with all RUSTs having the same outlook, ranging from ‘very positive’ to ‘very negative’ This figure was prepared with the R package *ggplot2*.

As a sensitivity analysis, we compare the summary effect sizes and their 95% confidence intervals under different scenarios. As was done for the Hpylori dataset in Worked Example 1 (Figure 3), we pretend that varying subsets of the studies in the greentea dataset were registered but never published; using the original dataset in Jurgens et al. [14], we set studies published from 2006 onwards as RUSTs and incrementally consider as published the studies in subsequent years, until reaching 2010. We then create a sensitivity plot of effect sizes as more studies are considered published. We examine the scenarios under which all RUSTs are assigned the same outlook, from ‘very positive’ to ‘very negative’. Figure 5 displays the variation in the estimated effect sizes as studies from 2006 onwards are considered published or not.

We can generate a forest plot for each of the ten outlooks in Table 2 with the option all.outlooks=TRUE.

> forestsens(greentea, binary=FALSE, mean.sd=TRUE, higher.is.better=FALSE, random.number.seed=52,**all.outlooks=TRUE**)

This command also generates a table of overall SMD’s, their confidence intervals (from the lower confidence limit lcl to the upper confidence limit ucl), and an estimate of τ^2^ (tau2), which is a measure of heterogeneity between studies (Table 6).

**Table 6:** **Summary effects for the** greentea **dataset after all unpublished studies assigned the same outlook**

**Scenario number**	**Outlook assigned to all 3 unpublished studies**	**Standardized mean difference (SMD) assigned to all unpublished studies**	**Summary effect**	**Lower 95% confidence limit**	**Upper 95% confidence limit**	***τ***^**2**^
1	very positive	−0.8	−0.411	−1.013	0.192	1.256
2	positive	−0.3	−0.519	−1.057	0.019	0.991
3	no effect	0	−0.586	−1.092	−0.080	0.868
4	negative	0.3	−0.651	−1.135	−0.166	0.791
5	very negative	0.8	−0.759	−1.230	−0.287	0.744
6	very positive CL	*LCL*_*pub,*1*-α*_	−0.877	−1.368	−0.386	0.810
7	positive CL	$0.5\left({\hat{\mathrm{SMD}}}_{\mathrm{pub}}+\mathrm{LC}L_{\mathrm{pub},1-\alpha }\right)$	−0.814	−1.290	−0.338	0.761
8	current	${\hat{\mathrm{SMD}}}_{\mathrm{pub}}$	−0.748	−1.219	−0.277	0.744
9	negative CL	$0.5\left({\hat{\mathrm{SMD}}}_{\mathrm{pub}}+\mathrm{UC}L_{\mathrm{pub},1-\alpha }\right)$	−0.681	−1.159	−0.204	0.767
10	very negative CL	*UCL*_*pub,*1*-α*_	−0.614	−1.110	−0.119	0.830

The random number seed used to generate this table was 52.

## Results and discussion

### Discussion of worked examples

The sensitivity analyses in the worked examples indicate that successful approximation of the actual summary effect depends on i) which outlooks the end-user selects (Tables 4 and 6) and ii) the ratio of number of published studies to RUSTs (Figures 3 and 5).

Figures 3 and 5 in the worked examples illustrate how close the estimated summary effect may come to the actual summary effect. This accuracy, however, depends largely on the end-user’s success in predicting the outcomes of registered, unpublished studies. As with any software, the ‘garbage in, garbage out’ principle applies to the use of SAMURAI. The end-user has the flexibility of choosing anticipated effect sizes and directions but also thereby assumes the burden of responsibility for making these choices.

#### Worked Example 1

By the year 2001, all 33 studies in the original dataset compiled by Ford et al. [13] had been published. As determined by the DerSimonian-Laird method, the summary relative risk was 0.66, and its 95% confidence interval was (0.58, 0.76) (Figure 3). The relative risk and its confidence interval are below 1.0, indicating that the proportion of patients not healed by the experimental intervention was lower than the proportion of patients not healed by the control intervention (recall that the events counted in this dataset represent numbers of patients not healed). Thus, the summary relative risk favors the experimental treatment.

Now, suppose it is the year 1998, when five of the studies remain unpublished. Table 4 shows that treating all studies published on or after 1998 as RUSTs with a ‘negative’ or ‘very negative’ outlook leads to an overall summary effect with a confidence interval that straddles a relative risk of 1.0, which corresponds to having no statistically significant difference between the two treatment arms beyond a 0.05 level. After changing the relative risk for a ‘negative’ study from the default value of 2 to 1.5, we see that having all unpublished studies with a ‘negative’ outlook no longer straddles a relative risk of 1.0. Thus, we see that the summary effect size is sensitive to the outlooks chosen by the end-user.

We can also see in Figure 3, that choosing a ‘very negative’ outlook would have suggested the non-significance of a treatment effect as early as 1998. Similarly, choosing a ‘negative’ outlook for all RUSTs would have suggested the non-significance of the treatment effect as early as 1997, when seven of the studies were unpublished.

Now suppose it is the year 1995, when 15 of these studies have been published while 18 studies yet remain as RUSTs, registered but unpublished. If we then choose to assign these 18 RUSTs the outlook ‘very negative’, then the summary relative risk and its 95% confidence interval would be above 1.0, thereby favoring the control intervention (as the proportion of patients not healed by the experimental intervention would exceed the proportion of patients not healed by the control intervention). If we instead elected to assign all 18 RUSTs the outlook ‘negative’, then the 95% confidence interval of the summary relative risk would straddle 1.0, suggesting there would be no statistically significant difference between the control and the experimental interventions. On the other hand, had we chosen ‘no effect’, ‘positive’, or ‘very positive’, then the summary relative risk and its 95% confidence interval would still be below 1.0, thereby favoring the experimental intervention. Thus, we see that the summary effect size is also sensitive to the ratio of published studies to RUSTs.

#### Worked Example 2

The summary treatment effect calculated for the original dataset compiled by Jurgens et al. [14] is –0.61, with a 95% confidence interval of (–1.10, –0.11). A standardized mean difference of zero corresponds to having no detectable difference between treatments. This result suggests that participants in the experimental arm lost more weight than participants in the control arm.

Table 6 shows that treating all studies published on or after 2009 as RUSTs with a ‘very positive’ or ‘positive’ outlook leads to an overall summary effect with a confidence interval that straddles an SMD of 0.0, which corresponds to having no statistically significant difference between the two intervention arms. In contrast, choosing any of the other eight outlooks does not result in a confidence interval that straddles 0.0. Thus, we see that the summary effect size is sensitive to the outlooks chosen by the end-user.

In Figure 5, we can see that successful approximation of the actual summary effect depends on which outlooks the end selects and on the ratio of number of published studies to RUSTs. Choosing a ‘very negative’ or ‘negative’ outlook would have suggested a statistically significant treatment effect until 2009, when only one RUST was left. Choosing a ‘no effect’ outlook would not have suggested a statistically significant treatment effect until 2008 when three RUSTs remained. In contrast, choosing a ‘positive’ or ‘very positive’ outlook would have suggested a statistically significant treatment effect at least as early as 2006.

### Comparison with existing methods and software

Out of all his numerous press conferences after 9/11, perhaps the best known quote by former US Secretary of Defense Donald Rumsfeld was this Socratic idea: “There are known knowns; there are things we know we know. We also know there are known unknowns; that is to say, we know there are some things we don’t know. But there are also unknown unknowns; the ones we don’t know we don’t know.”

Prior to the establishment of clinical trial registries, assessing the impact of unpublished studies on a meta-analysis has entailed making broad assumptions about the distribution of ‘unknown unknowns’. The trim and fill method by Duval & Tweedie [7] works under the assumption that the distribution of all effect sizes, published and unpublished, is symmetrical around the true mean. Selection models, such as the Copas selection model [8], assume that publication is conditionally dependent on effect size, i.e., the data is assumed to be missing at random.

The establishment of clinical trial registries now hold the promise of reducing the proportion of ‘unknown unknown’ effect sizes determined by unpublished studies and increasing the proportion of ‘known unknown’ effect sizes determined by RUSTs. However, this ideal is far from the current reality, for laws and regulations have not been thorough enough to ensure full and timely disclosure of completed trial results.

Discrepancies in reporting standards have allowed reporting bias to continue. Some data elements required by the ICMJE are optional for ClinicalTrials.gov and for the FDAAA of 2007, including study completion date and reasons why a study was stopped [15]. Also, the ICMJE requires registration prior to enrollment of the first participant, while ClinicalTrials.gov allows registration ‘at any time’, even after study completion. Ross et al. [16] found that out of a sample of registered trials completed prior to 2004, only 60% had their results published within 4 years. Mathieu et al. [17] found prevalence of selective reporting among adequately registered published studies, as evidenced by discrepancies between study outcomes registered and study outcomes published in 31% of those studies.

Furthermore, the increasing number of clinical trials being conducted outside of the US contributes to retrieval bias. FDAAA 801 only applies to ‘applicable clinical trials’, including trials with at least one site in the US. As clinical trials are increasingly held overseas, it is unclear how quickly the laws of other nations will catch up and require sufficient registration of these as well. The Trial and Experimental Studies Transparency Act of 2012 was introduced to the US Congress in May 2012 for the purpose of closing loopholes in the FDAAA; as of December 2013, it remains in committee, not having yet been approved for a vote on the House floor.

In the current landscape of clinical trial registries with its mix of ‘known unknowns’ and ‘unknown unknowns’, modeling may become too complicated to implement. Copas notes the limitations of selection models: “[P]ublication may depend on study size as well as many other features of the study’s design and outcome. However, attempts to fit more complicated selection models seem problematical, since when the number of studies is small (as is often the case in practice) the information in the data is very limited. No model will reflect all the reasons why some papers are selected and some are not” [18].

We have designed SAMURAI to incorporate enrollment information (required by FDAAA), but have forgone the theoretical rigor of previously existing methods in order to pursue the pragmatic goal of developing an exploratory tool for systematic reviewers (including non-statisticians) wishing to illustrate the potential, if not necessarily the most probable, impact of ‘known unknown’ RUSTs on a meta-analytic summary effect. That is, SAMURAI does not on its own conduct a probabilistic sensitivity analysis, but rather, it produces output that could be used in, say, a ‘best-case, worst-case’ analysis. As far as we know, there exists no other software that allows the end-user to include sample sizes of unpublished studies. However, we have largely left it up to the systematic reviewer as to how to address issues stemming from the unfulfilled potential of clinical trial registries.

We have designed SAMURAI on the premise that the requirements by the ICJME will eventually be incorporated into law. Thus we make the following assumptions: i) all studies will be registered before commencement; ii) all registered studies are easily accessible to the systematic reviewers; and iii) all results found by registered studies will be released in a timely manner. We thereby encourage systematic reviewers to use SAMURAI to make interim meta-analyses but not for drawing final conclusions. As noted by Deborah Zarin, director of ClinicalTrials.gov, “[J]ournals will continue to add value by publishing useful and readable trial reports that clinicians, the media, and patients can interpret and use… [T]he results disclosed for the FDA will not have been externally peer reviewed and will be preliminary” [19].

While the default methodology of SAMURAI is *ad hoc*, SAMURAI also allows end-users to impute effect sizes with values of their choosing rather than with the default values. The end-user may elect to use a model with assumptions about ‘unknown unknowns’ to generate these values; in so doing the end-user should be knowledgeable of the assumptions associated of that model.

While we hope that the consideration of RUSTs will, in the long term, sufficiently address publication bias, we acknowledge that the current reality is far from ideal. And while our approach leaves much of the burden of imputation up to the systematic reviewer, we hope that this burden will be lifted by regulations requiring more transparency on the part of study investigators.

No matter what approach is taken, the systematic reviewer should keep in mind that “high quality syntheses require considerably more than just the application of quantitative procedures” [16]. It is still incumbent upon systematic reviewers to examine clinical trials for other kinds of biases, such as trial designs that rig experimental treatment dosages to be much larger than control treatment dosages [20]. Thus, the estimates of confidence intervals presented in forest plots generated using SAMURAI should be regarded with some caution. As Copas notes, “If we see the aim of sensitivity analysis in terms of an informal warning of how sensitively the conclusions from a meta-analysis can depend on selection, then, arguably, the numerical accuracy of these intervals for specific values of *P* <1 is not particularly important” [18]. After all, ‘known unknowns’ are still unknowns.

### Assumptions and limitations

Pigott differentiates between the following types of missing data: missing studies, missing effect sizes, and missing covariates [21]. SAMURAI handles missing effect sizes under the premise that all relevant missing studies have been identified. SAMURAI does not handle missing covariates, but rather assumes that a random effects model without covariates adequately accounts for variation between studies.

Some additional assumptions were listed in the Methods section. As a result, the justification or utility of the use of SAMURAI may be weakened in cases where i) there exist a large number of unpublished and unregistered trials, ii) event rates of control arms of unpublished studies with binary outcomes may be substantially different from the event rate across the control arms of published studies, or iii) heterogeneity among studies is severe enough to question whether stratification of studies would be more appropriate (an easy workaround for severe heterogeneity, however, may be to compute a summary effect for each strata).

DerSimonian and Kacker [22] have proposed alternative random effects modeling approaches as improvements upon the commonly used DerSimonian-Laird method. Future software could implement the more complex but less widespread methods they proposed.

## Conclusions

With the increase in the number of registered clinical trials, systematic reviewers are now more likely to acquire evidence of the existence of unpublished studies. However, they have not yet had a statistical approach to incorporate this information into quantitative analyses. SAMURAI could prove a useful tool for reviewers to integrate information from unpublished studies to assess the potential impact of publication bias on the results of meta-analyses.

### Availability and requirements

**Project name:** SAMURAI

**Project home page:**http://cran.r-project.org/web/packages/SAMURAI/index.html

**Operating system(s):** Platform independent

**Programming language:** R

**License:** GNU GPL 2 or 3

### **Package installation**

We have written SAMURAI as an R package, and have made the SAMURAI package available free of charge under an open source General Public License (GPL). Users of SAMURAI will also need to install and load the existing R package metafor [11].

Using R GUI or RStudio, one can install these packages with the following commands:

> install.packages(‘metafor’)

> install.packages(‘SAMURAI’)

Alternatively, one may download the SAMURAI and metafor packages from the Comprehensive R Archive Network (CRAN) at the following websites:

http://cran.r-project.org/web/packages/SAMURAI/index.html,

http://cran.r-project.org/web/packages/metafor/index.html.

## Abbreviations

FDAAA: Food and Drug Administration Amendments Act; ICMJE: International Committee of Medical Journal Editors; RUST: Registered unpublished study; SMD: Standardized mean difference.

## Competing interests

The authors declare that they have no competing interests.

## Authors’ contributions

NYK, conception and design, data collection and analysis, manuscript writing, critical revision, final approval of the manuscript. SIB, conception and design, data collection and analysis, manuscript writing, critical revision, final approval of the manuscript. KT, conception and design, critical revision, final approval of the manuscript. GG, conception and design, data collection and analysis, manuscript writing, critical revision, final approval of the manuscript.

## Supplementary Material

Additional file 1 Technical Appendix for the R Package SAMURAI. Click here for file
