# Supplementary material for: SAMURAI: Sensitivity analysis of a meta-analysis with unpublished but registered analytical investigations (software)
Source: Syst Rev. 2014 Mar 18;3:27. doi: 10.1186/2046-4053-3-27 (PMC4021727; doi:10.1186/2046-4053-3-27)
Supplement: Additional file 1 — Technical Appendix for the R Package SAMURAI. [file 2046-4053-3-27-S1.pdf]

Technical Appendix for the R Package `SAMURAI`

Last updated: April 2014.

1 Formatting a data set file for use by `SAMURAI`

1.1 Binary outcomes

The data set should have the same column headings as those in the example below in Table 1.

Table 1: An example of a data set with studies with binary outcomes.

number	study	year	outlook	ctrl.n	ctrl.events	expt.n	expt.events
1	P1	2001	published	60	15	60	9
2	P2	2002	published	75	19	75	12
3	P3	2003	published	50	14	40	5
4	U1		negative	50		100	
5	U2		no effect	75		75	

The `outlook` of a study can be one of the following: "published", "very positive", "positive", "no effect", "negative", "very negative", "very positive CL", "positive CL", "current effect", "negative CL", or "very negative CL".

`ctrl.n` and `expt.n` refer to the sample sizes of the control and experimental arms, respectively.

`ctrl.events` and `expt.events` refer to the numbers of events within the control and experimental arms, respectively.

1.2 Continuous outcomes

A data set with continuous outcomes can be in one of two formats.

1.2.1 Means and standard deviations

For studies containing the mean effect and its standard deviation for each arm of a published study, the column headings should be the same as in the example below in Table 2.

Table 2: An example of a data set with means and standard deviations for each treatment arm.

number	study	year	outlook	ctrl.n	ctrl.mean	ctrl.sd	expt.n	expt.mean	expt.sd
1	P1	2001	published	40	92	20	40	94	12.9
2	P2	2002	published	50	88	26	50	82.20	11.3
3	P3	2003	published	60	82	22	60	0.90	25.1
4	U1		negative	50			50		
5	U2		no effect	75			75		

`ctrl.n` and `expt.n` refer to the sample sizes of the control and experimental arms, respectively.

`ctrl.mean` and `expt.mean` refer to the mean effect size within the control and experimental arms, respectively.

`ctrl.sd` and `expt.sd` refer to the standard deviation of the effect size within the control and experimental arms, respectively.

1.2.2 Standardized mean differences (SMD)

For studies containing data on the standardized mean difference (SMD) and its variance for each published study, the column headings should be the same as in the example below in Table 3. The SMD should be equivalent to Hedges' g.

Table 3: An example of a table with standardized mean differences (SMD).

number	study	year	outlook	ctrl.n	expt.n	smd	smd.v
1	P1	2001	published	60	60	0.095	0.033
2	P2	2002	published	65	65	0.277	0.031
3	P3	2003	published	40	40	0.367	0.05
4	U1		negative	100	100		
5	U2		no effect	75	75		

`ctrl.n` and `expt.n` refer to the sample sizes of the control and experimental arms, respectively. `smd` is the SMD and `smd.v` is its variance.

2 Importing a data set file

The R function `read.csv()` can be used to import CSV files that separate values by commas.

The R function `read.csv2()` can be used to import CSV files that separate values by semi-colons (as is done on computers with Microsoft Windows with German language settings).

3 Pseudocode of `forestsens()`

Step 0: (Optional) Designate all unpublished studies to have the same outlook (i.e. the same risk ratio). The user can override the outlooks of the unpublished studies with a specified outlook by using the option `outlook`. For example, to assign the outlook "no effect" to all unpublished studies, we can specify the option `outlook=‘‘no effect’’`.

3.1 For studies with binary outcomes

Step B1: Subset the published studies. Calculate the log risk ratio and its variance for each of the published studies. Calculate a summary effects across the collectoin of published studies using a random effects model. Let k denote the number of published studies included in the meta-analysis. For the j-th published study, with $j \in \{1, \dots, k\}$, denote x_{0j} events out of n_{0j} persons in the control group, and x_{1j} events out of n_{1j} persons in the treatment group.

Table 4: Published studies

	Intervention		Control	
Study	Events	Sample size	Events	Sample size
Published study 1	x_{11}	n_{11}	x_{01}	n_{01}
$\vdots$	$\vdots$	$\vdots$	$\vdots$	$\vdots$
Published study k	x_{1k}	n_{1k}	x_{0k}	n_{0k}
Totals over published studies	$A = \sum_{j=1}^{k} x_{1j}$	$n_1 = \sum_{j=1}^{k} n_{1j}$	$C = \sum_{j=1}^{k} x_{0j}$	$n_0 = \sum_{j=1}^{k} n_{0j}$

Step B2: For each individual published study Let $\hat{p}_{0j} = x_{0j}/n_{0j}$ be the estimate of the rate of events in the control group, and $\hat{p}_{1j} = x_{1j}/n_{1j}$ be the estimate of the rate of events in the treatment group. Calculate the estimate of the log risk ratio as

$$\log \widehat{RR}_j = \log\left(\hat{p}_{1j}/\hat{p}_{0j}\right) = \log\left(\frac{x_{1j}/n_{1j}}{x_{0j}/n_{0j}}\right)$$

and its approximate variance as

$$\widehat{var}\left(\log \widehat{RR}_j\right) = \frac{1}{x_{0j}} + \frac{1}{x_{1j}} - \frac{1}{n_{0j}} - \frac{1}{n_{1j}}.$$

The lower and upper bounds of the $(1-\alpha)$ confidence interval (with $\alpha \in [0,1]$) of the risk ratio are then defined to be

$$LCL_{j,1-\alpha} = \exp\left\{\log \widehat{RR}_j - z_{1-\alpha/2}\sqrt{\widehat{var}\left(\log \widehat{RR}_j\right)}\right\},$$

$$UCL_{j,1-\alpha} = \exp\left\{\log \widehat{RR}_j + z_{1-\alpha/2}\sqrt{\widehat{var}\left(\log \widehat{RR}_j\right)}\right\},$$

where $z_{1-\alpha/2}$ is selected such that for a standard normal random variable Z, $P(|Z| > z_{1-\alpha/2}) = 1-\alpha$. The default confidence level is 95%.

Step B3: For the published studies collectively To get a summary effect of the published studies using a random effects model, the binary outcome data are converted to log risk ratios. (See Step B2.) Then a summary effect is calculated using a random effects model via the DerSimonian & Laird method [DS86; BHHR09]. This is accomplished using the R package `metafor` function `rma()` with the option `method=DL`. The default confidence level is 95%.

DerSimonian & Laird method The steps of the DerSimonian & Laird method are as follows:

1. For each study, calculate the estimate of the variance $\hat{v}_j = \widehat{var}\left(\log \widehat{RR}_j\right)$. (See Step B2.)

2. Estimate the between-studies variance τ^2 as follows: Weight each study by the inverse of the variance. $w_j = 1/\hat{v}_j$. Add up the weights. $W = \sum_{j=1}^{k} w_j$. Also calculate the following quantities: $W2 = \sum_{j=1}^{k} w_j^2$; $WY = \sum_{j=1}^{k} w_j y_j$, $WY2 = \sum_{j=1}^{k} w_j y_j^2$, where y_j is the standardized mean difference in the j-th study. Then an estimator of τ^2 is:

$$\hat{\tau}^2 = \frac{WY2 - \frac{(WY)^2}{W} - (k-1)}{W - \frac{W2}{W}}.$$

3. For each study, define the total variance as $v_j + \tau^2$. Weight each study by the inverse of the estimated total variance. $w_j^* = 1/(\hat{v}_j + \hat{\tau}^2)$. (Note that the between-studies variance and the within-studies variances are assumed to be independent of each other.) Add up these weights. $W^* = \sum_{j=1}^{k} w_j^*$. Also calculate $W^*Y = \sum_{j=1}^{k} w_j^* y_j$. Then a random-effects model effect for the summary log risk ratio is $M^* = W^*Y/W^*$, and its approximate variance is $V_{M^*} = 1/W^*$.

Then the estimate of the summary log risk ratio is

$$\log \widehat{RR}_{pub} = W^*Y/W^*,$$

and its approximate variance as

$$\widehat{var}\left(\log \widehat{RR}_{pub}\right) = 1/W^*.$$

The lower and upper bounds of the $(1-\alpha)$ confidence interval (with $\alpha \in [0,1]$) of the summary risk ratio are then defined to be

$$LCL_{pub,1-\alpha} = \exp\left\{\log \widehat{RR}_{pub} - z_{1-\alpha/2}\sqrt{\widehat{var}\left(\log \widehat{RR}_{pub}\right)}\right\},$$
$$UCL_{pub,1-\alpha} = \exp\left\{\log \widehat{RR}_{pub} + z_{1-\alpha/2}\sqrt{\widehat{var}\left(\log \widehat{RR}_{pub}\right)}\right\},$$

where $z_{1-\alpha/2}$ is selected such that for a standard normal random variable Z, $P(|Z| > z_{1-\alpha/2}) = 1-\alpha$.

Detailed examples can be found in Chapter 14 of Borenstein, Hedges, Higgins, & Rothstein [BHHR09]. Those examples involve log odds ratios but the procedure is the same for log risk ratios.

Step B4: For the unpublished studies, impute the number of events in the control arms, based on the risk of events in the control arms of the published studies. No random variation is used to impute these numbers. We assume that the rates of events in the control arms are the same across all published and unpublished studies. Let m denote the number of unpublished studies included in the meta-analysis, and let $\hat{p}_{0,pub} = C/n_0$ denote the estimated proportion of events across the control arms of all published studies. For the i-th unpublished study ($i \in \{1,\ldots,m\}$), with n_{0i} persons in the control arm (and n_{1i} persons in the treatment arm), impute the number of events within the control arm to be

$$x_{0i} = [n_{0i}\hat{p}_{0,pub}],$$

that is, $n_{0i}\hat{p}_{0,pub}$ rounded to the nearest integer. Repeat for all m unpublished studies.

Step B5: Assign risk ratios to all defined outlooks. Defined outlooks include outlooks based on the confidence interval of the log risk ratio of the published studies. The default risk ratios assigned to each of the outlooks are as follows, depending on whether the event is desirable or not.

Table 5: Default risk ratios assigned to unpublished studies

Outlook	`higher.is.better=TRUE`	`higher.is.better=FALSE`
very positive	3	0.33
positive	2	0.5
no effect	1	
negative	0.5	2
very negative	0.33	3
very positive CL	$UCL_{pub,0.95}$	$LCL_{pub,0.95}$
positive CL	$0.5(\widehat{RR}_{pub} + UCL_{pub,0.95})$	$0.5(\widehat{RR}_{pub} + LCL_{pub,0.95})$
current effect	$\widehat{RR}_{pub}$	
negative CL	$0.5(\widehat{RR}_{pub} + LCL_{pub,0.95})$	$0.5(\widehat{RR}_{pub} + UCL_{pub,0.95})$
very negative CL	$LCL_{pub,0.95}$	$UCL_{pub,0.95}$

Note that outlooks denoted with "CL" are defined according to a confidence interval of the risk ratio of the published studies collectively. (See Step B3.)

Step B6: For each of the unpublished studies, estimate the proportion of events in the intervention arm, based on the risk ratio assigned to the study outlook. Then impute the number of events in the intervention arm randomly from a binomial distribution. Let $m_t \leq m$ be the number of unpublished studies in the meta-analysis with outlook t, and denote the proportion of events in the treatment and control arms of such studies as p_{1t} and p_{0t} respectively. Note that $\sum_t m_t = m$. Extract the assigned risk ratio $RR_t = p_{1t}/p_{0t}$ for studies with outlook t from Table 5. Estimate the proportion of events in the treatment arms by rearranging the formula for risk ratio:

$$\hat{p}_{1t} = RR_t \cdot \widehat{p}_{0t} = RR_t \cdot \widehat{p}_{0,pub}.$$

(Note that $\hat{p}_{0t} = \widehat{p}_{0,pub}$ since we have assumed that the true rate of event in the control arm is the same across all studies.)

For the i-th unpublished study, having outlook t and treatment sample size n_{1i}, impute the number of events within the treatment arm to be $x_{1i} = [X]$, where X is a random variable from a binomial distribution with mean $y_i = n_{1i}\hat{p}_{1t}$ and variance $v_i = \hat{p}_{1t}(1 - \hat{p}_{1t})/n_{1i})$.

Step B7: For each unpublished study, calculate the standard error of the log risk ratio using the imputed figures for the numbers of events in the control and intervention arms. Calculate a summary effect across the collection of unpublished studies using a random effects model. Calculate a summary effect across all (published and unpublished) studies in the meta-analysis using a random effects model. These summary effects are calculated using the DerSimonian & Laird method, as detailed under Step B3.

Step B8: Graph a forest plot of individual and aggregate results. This is done using the `metafor` functions `forest()` and `addpoly()` [WV10].

Step B9: (Optional) Repeat Steps B1 to B8 for each outlook. The user can generate one plot for each of the ten outlooks defined for unpublished studies (see Table 5) by using the option `all.outlooks=TRUE`.

3.2 For studies with continuous outcomes

Step C1: If the data is in the form of means and standard deviations, convert the data in each study to a standardized mean difference (Hedges' g). The following procedure is from [BHHR09].

The following formula for pooled within-groups standard deviation is used:

$$s_{within} = \sqrt{\frac{(n_{ctrl} - 1)s^2_{ctrl} + (n_{expt} - 1)s^2_{expt}}{n_{ctrl} + n_{expt} - 2}}$$

This is used to estimate Cohen's d:

$$d = \frac{\overline{x}_{expt} - \overline{x}_{ctrl}}{s_{within}}$$

Cohen's d can be converted into Hedges' g using the conversion factor

$$J = 1 - \frac{3}{4(n_{ctrl} + n_{expt} - 2) - 1}.$$

Hedges' g is then

$$g = Jd.$$

Step C2: Subset the published studies. Impute the variance of the SMD of each study using a 'very good' approximator mentioned by Borenstein [CHV09, 226]. Calculate the summary SMD for the published studies with a random-effects model using the method by DerSimonian & Laird [DS86]. Let k denote the number of published studies included in the meta-analysis, indexed by $j \in \{1, \ldots, k\}$.

Table 6: Published studies

Study	Standardized mean difference (SMD)	Sample size	
		Intervention	Control
Published study 1	y_1	n_{11}	n_{01}
⋮	⋮	⋮	⋮
Published study k	y_k	n_{1k}	n_{0k}
Totals over published studies	–	$n_1 = \sum_{j=1}^{k} n_{1j}$	$n_0 = \sum_{j=1}^{k} n_{0j}$

Step C3: For each published study Imputing the variance of the SMD can be done as follows if we assume the SMD is equivalent to Hedges' g: Let $\nu = k - 1$ and $J_\nu = 1 - 3/(4\nu - 1)$. J_ν is known as a correction factor for converting Cohen's d to Hedges' g. Assuming the SMD is equivalent to Hedges' g, convert the SMD to Cohen's d using the formula $d = g/J_\nu$. Then use a 'very good' approximator of the variance v_d of Cohen's d mentioned by Borenstein [CHV09, 226]:

$$\hat{v}_d = \frac{n_1 + n_0}{n_1 n_0} + \frac{d^2}{2(n_1 + n_2)},$$

where n_0, n_1 are as defined in Table 6.

The variance v_g of Hedges' g is then approximated by the following:

$$\hat{v}_g = J_\nu^2 \hat{v}_d.$$

Then define the lower and upper bounds of the $(1-\alpha)$ confidence interval (with $\alpha \in [0, 1]$) of the SMD as

$$LCL_{i,1-\alpha} = \exp\left\{ y_j - z_{1-\alpha/2}\sqrt{\hat{v}_g} \right\},$$
$$UCL_{i,1-\alpha} = \exp\left\{ y_j + z_{1-\alpha/2}\sqrt{\hat{v}_g} \right\},$$

where y_j denotes the SMD of the j-th study, and $z_{1-\alpha/2}$ is selected such that, for a standard normal random variable Z, $P(|Z| > z_{1-\alpha/2}) = 1 - \alpha$.

Step C4: For the published studies collectively To get a summary effect of the published studies using a random effects model, use the DerSimonian-Laird method [DS86; BHHR09] to calculate y_{pub}, the standardized mean difference (SMD) across all published studies. This is accomplished using the R package `metafor` function `rma()` with the option `method=DL`. The default confidence level is 95%.

DerSimonian & Laird method The steps of the DerSimonian & Laird method are as the same as under Step B3, except as follows:

1. For each study, calculate the estimate of the variance of the SMD: $\hat{v}_j = \widehat{var}\,(y_j)$, where y_j is the standardized mean difference in the j-th study. (See Step C3.)

Step C5: Assign SMD to all defined outlooks. Defined outlooks include outlooks based on the confidence interval of the SMD of the published studies collectively. The default risk ratios assigned to each of the outlooks are as follows, depending on whether the event is desirable or not.

Table 7: Default SMD assigned to unpublished studies

Outlook	`higher.is.better=TRUE`	`higher.is.better=FALSE`
very positive	0.8	-0.8
positive	0.3	-0.3
no effect	0	
negative	-0.3	0.3
very negative	-0.8	0.8
very positive CL	$UCL_{pub,0.95}$	$LCL_{pub,0.95}$
positive CL	$0.5(\widehat{SMD}_{pub} + UCL_{pub,0.95})$	$0.5(LCL_{pub,0.95} + \widehat{SMD}_{pub})$
current effect	$\widehat{SMD}_{pub}$	
negative CL	$0.5(LCL_{pub,0.95} + \widehat{SMD}_{pub})$	$0.5(\widehat{SMD}_{pub} + UCL_{pub,0.95})$
very negative CL	$LCL_{pub,0.95}$	$UCL_{pub,0.95}$

Note that outlooks denoted with "CL" are defined according to a confidence interval of the risk ratio of the published studies collectively. (See Step C4.)

Step C6: For each unpublished study, impute the SMD and its variance. Based on the outlook of the unpublished study, impute the SMD using Table 7. Then employ Borenstein's 'very good' approximator of v_d (as we did in Step C3).

Step 7: Calculate a summary effect across the collection of unpublished studies using a random effects model. Calculate a summary effect across all (published and unpublished) studies in the meta-analysis using a random effects model. These summary effects are calculated using the DerSimonian & Laird method, as detailed under Step C4.

Step 8: Graph a forest plot of individual and aggregate results. This is done using the `metafor` functions `forest()` and `addpoly()` [WV10].

Step 9: (Optional) Repeat Steps C1 to C8 for each outlook. The user can generate one plot for each of the ten outlooks defined for unpublished studies (see Table 7) by using the option `all.outlooks=TRUE`.

References

1. [BHHR09] Borenstein, Hedges, Higgins, & Rothstein. *Introduction to Meta-analysis.* UK: Wiley, 2009.

2. [CHV09] Cooper, Hedges, & Valentine, eds. *Handbook of Research Synthesis & Meta-analysis*, 2nd ed. NY: Russell Sage Foundation, 2009.

3. [DL86] Rebecca DerSimonian and Nan Laird. "Meta-analysis in clinical trials." *Controlled Clinical Trials* 7:177-188 (1986).

4. [WV10] Wolfgang Viechtbauer (2010). "Conducting Meta-Analyses in R with the `metafor` Package." *Journal of Statistical Software*, 36:3 (August 2010).
